# Singing activity‐driven *Arc* expression associated with vocal acoustic plasticity in juvenile songbird

**DOI:** 10.1111/ejn.14057

**Published:** 2018-07-06

**Authors:** Shin Hayase, Kazuhiro Wada

**Affiliations:** ^1^ Graduate School of Life Science Hokkaido University Sapporo Hokkaido Japan; ^2^ Department of Biological Sciences Hokkaido University Sapporo Hokkaido Japan; ^3^ Faculty of Science Hokkaido University Sapporo Hokkaido Japan

**Keywords:** immediate early gene, learning efficiency, motor learning, sensitive period

## Abstract

Learned vocalization, including birdsong and human speech, is acquired through self‐motivated vocal practice during the sensitive period of vocal learning. The zebra finch (*Taeniopygia guttata*) develops a song characterized by vocal variability and crystallizes a defined song pattern as adulthood. However, it remains unknown how vocal variability is regulated with diurnal singing during the sensorimotor learning period. Here, we investigated the expression of activity‐dependent neuroplasticity‐related gene *Arc* during the early plastic song phase to examine its potential association with vocal plasticity. We first confirmed that multiple acoustic features of syllables in the plastic song were dramatically and simultaneously modulated during the first 3 hr of singing in a day and the altered features were maintained until sleep. In a concurrent manner, *Arc* was intensely induced during morning singing and a subsequent attenuation during afternoon singing in the robust nucleus of the arcopallium (RA) and the interfacial nucleus of the nidopallium (NIf). The singing‐driven *Arc* expression was not altered by circadian rhythm, but rather reduced during the day as juveniles produced more songs. Song stabilization accelerated by testosterone administration in juveniles was accompanied with attenuation of *Arc* induction in RA and NIf. In contrast, although early‐deafened birds produced highly unstable song even at adulthood, singing‐driven *Arc* expression was not different between intact and early‐deafened adults. These results suggest a potential functional link between *Arc* expression in RA and NIf and vocal plasticity during the sensorimotor phase of song learning. Nonetheless, *Arc* expression did not reflect the quality of bird's own song or auditory feedback.

**Abbreviations**
A: arcopallium
AMPAR: α‐amino‐3‐hydroxy‐5‐methyl‐4‐isoxazolepropionic acid receptor
Arc: activity‐regulated cytoskeleton‐associated protein
Area X: Area X of the striatum
d/v/a/p: dorsal/ventral/anterior/posterior
DLM: medial nucleus of dorsolateral thalamus
FM: frequency modulation
H: hyperpallium
HVC: (used as a proper name)
LMAN: lateral magnocellular nucleus of the anterior nidopallium
LTD: long‐term depression
LTP: long‐term potentiation
M: mesopallium
NIf: the nucleus interface of the nidopallium
NMDAR: *N*‐methyl‐d‐aspartate receptor
N: nidopallium
nXIIts: the tracheosyringeal part of the 12th cranial nerve nuclei
phd: posthatching day
P: pallidum
RA: the robust nucleus of the arcopallium
St: striatum
Th: thalamus
T: testosterone

## 1 INTRODUCTION

Complex motor skills, such as human speech, playing instruments and birdsong, are acquired through sensorimotor learning via repeated and self‐motivated motor practice (Bengtsson et al., 2005; Doupe & Kuhl, 1999; Elbert, Pantev, Wienbruch, Rockstroh, & Taub, 1995; Snow & Hoefnagel‐Höhle, 1978). Practice occurs at a steady rate throughout the day, but the largest improvement in performance occurs during the first hours of the day, suggesting that there is no simple relationship between the accumulation of motor practice and skill improvement during the learning period. This phenomenon is observed in many animal species, including primates, rodents and songbirds (Buitrago, Schulz, Dichgans, & Luft, 2004; Cao et al., 2015; Deregnaucourt, Mitra, Feher, Pytte, & Tchernichovski, 2005; Malone, Vasudevan, & Bastian, 2011; Robinson, Soetedjo, & Noto, 2006; Yin et al., 2009; Zhou, Weldon, Tang, & King, 2003). Nevertheless, it remains unknown how motor skill improvement is associated with diurnal repeated practice during the learning period.

**Figure 1:**
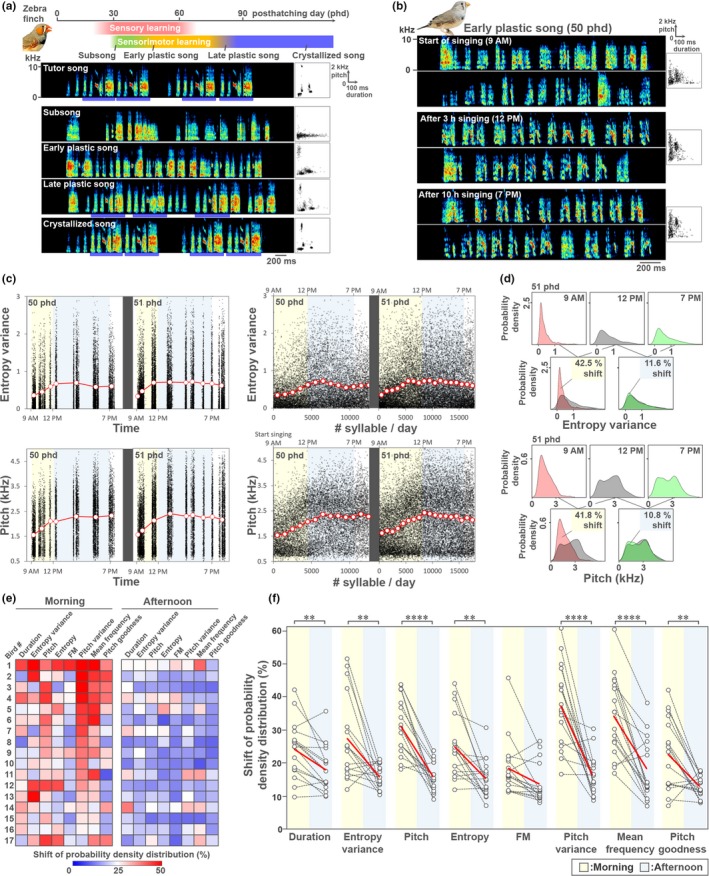
Diurnal shift and stabilization of song syllable acoustics during the early plastic song stage in the zebra finch. (a) The critical period of song learning (upper panel) and song development (lower panels) in the zebra finch. Blue bars in the lower panels represent the motif structure of song. Scatter plots indicate 500 syllables distribution (duration vs. pitch). (b) Early plastic songs of a zebra finch juvenile (50 phd) in the morning (9 a.m.), afternoon (12 p.m.) and evening (7 p.m.) in a day. Two song examples are represented at each time point. (c) Trajectory plots of entropy variance and pitch of all song syllables during two successive days produced by the same bird shown in (b) (12,506 syllables at 50 phd; 17,845 syllables at 51 phd). Acoustic features were plotted against time (left panels) or the order of syllables (right panels). Red‐lined circles indicate the average of each song cluster (left panels) or each 1,000 syllables (right panels). (d) Distribution of probability density of entropy variance and pitch in the morning (9 a.m.), afternoon (12 p.m.) and evening (7 p.m.) using 500 syllables at each time point (upper panels). Comparison of probability density of the two acoustic features for assessment of the acoustic shifts (%) during morning and afternoon periods (lower panels). (e) Individual differences of acoustic shifts (%) during morning and afternoon periods for eight acoustic features (duration, entropy variance, pitch, entropy, FM, pitch variance, mean frequency and pitch goodness). *n* = 17 birds. (f) Comparison of the acoustic shifts (%) between morning and afternoon periods during the early plastic song stage (45–53 phd, *n* = 17). Black lines indicate acoustics shifts of individual bird. Red lines indicate average of 17 birds. *p <* *0.05, **p <* *0.01, ***p <* *0.001, ****p <* *0.0001; mixed‐model ANOVA with Bonferroni correction. [Colour figure can be viewed at http://wileyonlinelibrary.com]

Juvenile male zebra finches develop their song from highly variable vocalizations to achieve the stereotyped acoustic structures of adult crystallized song (Figure 1a) (Immelmann, 1969; Price, 1979). Song acquisition is achieved through thousands of self‐motivated singing utterances during the critical period of vocal learning (Johnson, Soderstrom, & Whitney, 2002; Ohgushi, Mori, & Wada, 2015). The acoustic features of song syllables are highly variable during the early plastic song phase (40–60 posthatching days [phd]), compared with those in the crystallized song produced at adulthood (Deregnaucourt et al., 2004; Wood, Osseward, Roseberry, & Perkel, 2013). Furthermore, during a single day in the early plastic song phase, the average of syllable acoustics, such as entropy variance, greatly shifts in the morning when compared with the afternoon (Deregnaucourt et al., 2005; Shank & Margoliash, 2009), indicating diurnal regulation of vocal plasticity.

**Figure 2:**
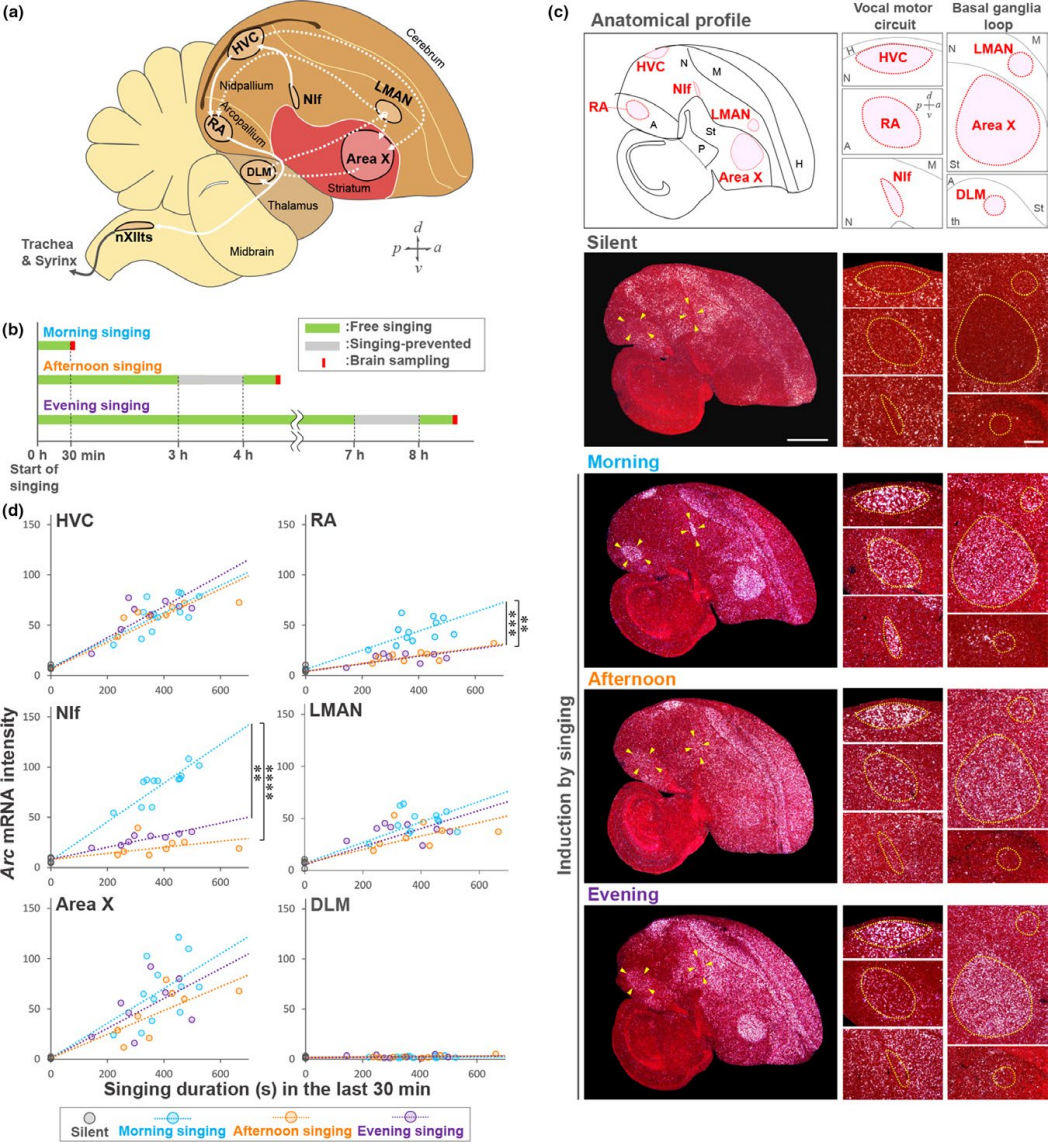
Diurnal change in *Arc* induction rates in RA and NIf during singing of the early plastic song phase. (a) Diagram of the neural circuits for song learning and production. The vocal motor circuit and the anterior forebrain pathway (pallial–basal ganglia–thalamic loop circuit) are represented as white solid and dotted lines, respectively. HVC (used as a proper name); RA, the robust nucleus of the arcopallium; Area X, Area X of the striatum; DLM, dorsal lateral nucleus of the medial thalamus; LMAN, lateral magnocellular nucleus of the anterior nidopallium; NIf, interfacial nucleus of the nidopallium; nXIIts, the tracheosyringeal part of the 12th cranial nerve nuclei. (b) Experimental timeline of brain sampling for morning, afternoon and evening singing. (c) Typical examples of expression of *Arc* mRNA in silence and singing during morning, afternoon and evening periods (47–52 phd). Singing duration (s) in 30 min after the initiation of singing at each time point is shown at the bottom. White signals: *Arc* mRNA. Red: Cresyl violet counter‐stained cells. Sections are sagittal. Scale bar = 1.5 mm. (right panel) Higher magnification images of *Arc* expression in song nuclei. A, arcopallium; H, hyperpallium; M, mesopallium; N, nidopallium; P, pallidum; St, striatum. Scale bar = 200 μm. (d) Induction rate of *Arc* mRNA in song nuclei during singing in the morning (light blue; *n* = 12 birds, 45–54 phd, mean = 48.7 phd), afternoon (orange; *n* = 8 birds, 43–60 phd, mean = 51.8 phd) and evening (purple; *n* = 8 birds, 45–55 phd, mean = 50.5 phd). Silent condition (black; *n* = 5 birds, 50–55 phd, mean = 53.4 phd). **p <* *0.001, ***p <* *0.0001, ****p <* *0.00001; ANCOVA with Bonferroni correction. [Colour figure can be viewed at http://wileyonlinelibrary.com]

For song learning and production, songbirds possess specialized neural circuits composed of a set of brain areas called the song nuclei: the vocal motor pathway necessary for song production and the anterior forebrain pathway (AFP) that is important for song learning (Figure 2a) (Bottjer, Miesner, & Arnold, 1984; Nottebohm, Stokes, & Leonard, 1976; Scharff & Nottebohm, 1991). The AFP forms a pallial–basal ganglia–thalamic loop composed of three song nuclei, that is the lateral magnocellular nucleus of the anterior nidopallium (LMAN), the basal ganglia nucleus Area X and the medial nucleus of the dorsolateral thalamus (DLM). HVC (proper name) projects to both Area X in the AFP and RA in the motor pathway. The vocal motor nucleus RA, which is analogous to the mammalian motor cortex, projects to the tracheosyringeal part of the 12th cranial nerve nuclei (nXIIts) that connects to syringeal muscles (Figure 2a) (Pfenning et al., 2014; Vicario & Nottebohm, 1988; Wild, 1993). During singing, RA integrates time‐locked input from HVC and the basal ganglia loop activity from LMAN and then outputs bursting activity to nXIIts, which constructs the syllable acoustics (Aronov, Andalman, & Fee, 2008; Fee, Kozhevnikov, & Hahnloser, 2004; Kao, Doupe, & Brainard, 2005; Sober, Wohlgemuth, & Brainard, 2008). NIf is the main nucleus providing auditory input to the song system via HVC in adult male zebra finches (Cardin & Schmidt, 2004; Coleman & Mooney, 2004) and shows premotor activity during song production (McCasland, 1987; Naie & Hahnloser, 2011; Vyssotski, Stepien, Keller, & Hahnloser, 2016).

At the molecular level, a large variety of genes are developmentally regulated in the song nuclei during the critical period of song learning in the zebra finch (Mori & Wada, 2015; Olson, Hodges, & Mello, 2015). Some immediate‐early genes are differentially induced by singing in the song nuclei between juvenile and adult stages (Jin & Clayton, 1997; Wada et al., 2006). The activity‐regulated cytoskeleton‐associated protein *Arc* (also called *Arg3.1*) is a neuronal activity‐dependent effector of long‐term potentiation and long‐term depression (LTD), via regulation of the endocytic trafficking of AMPA glutamate receptors (AMPARs) in dendritic spines, indicating a critical regulation of synaptic plasticity (Chowdhury et al., 2006; Messaoudi et al., 2007; Plath et al., 2006; Shepherd & Bear, 2011; Steward, Wallace, Lyford, & Worley, 1998). Although *Arc* exhibits song‐related expression in neurons within the zebra finch song nuclei and auditory regions (Lin, Vanier, & London, 2014; Velho, Pinaud, Rodrigues, & Mello, 2005; Wada et al., 2006), the relationship between vocal plasticity and *Arc* expression in the song nuclei has not been elucidated.

In this study, we investigated a potential association between singing‐driven *Arc* induction and vocal plasticity during the early plastic song phase. We first confirmed that syllable acoustics greatly shift during the first 3 hr of singing in a day. In a concurrent manner, changes in diurnal singing behaviour patterns were also associated with *Arc* expression, as singing‐induced intense *Arc* expression in RA and NIf in the morning but not the afternoon. *Arc* expression was attenuated when juveniles accumulated diurnal singing, but not regulated by circadian rhythm. To examine the modification of *Arc* expression by song quality or auditory feedback, we tested singing‐driven *Arc* expression in the testosterone (T)‐implanted juveniles that generated stabilized songs and in early‐deafened adults that produced highly variable songs. These results suggest that singing‐driven *Arc* expression associates with the acoustic stability of syllables specifically in the early plastic song phase.

## 2 MATERIALS AND METHODS

### 2.1 Animals

Male zebra finches were obtained from our breeding colonies at Hokkaido University. Birds were kept in breeding cages under a 13:11‐hr light/dark cycle. Light‐on was set at 8:30 a.m. We used the birds that started singing within 30 min after lights on for all experiments and analyses. During song recording sessions or singing prevention, each bird was individually housed in a cage inside a sound‐attenuating box (400 × 470 × 500 mm). They had ad libitum access to water and food. All experiments were conducted under the guidelines and approval of the Committee on Animal Experiments of Hokkaido University. These guidelines are based on the national regulations for animal welfare in Japan (Law for the Humane Treatment and Management of Animals with partial amendment No. 105, 2011).

### 2.2 Song recording and analysis

Songs were recorded using a unidirectional microphone (SM57, Shure, IL, USA) connected to a computer with the sound‐event triggered recording software sound analysis pro (sap v2011.089; http://soundanalysispro.com/) (Tchernichovski, Nottebohm, Ho, Pesaran, & Mitra, 2000). Each song bout was saved as a sound file (wav file), including time information. Low‐frequency noise (<0.5 kHz) and mechanical noise were filtered out using avisoft‐SASLab (Avisoft Bioacoustics, Glienicke, Germany). Analysis of syllables acoustic features was performed using SAP program while measuring eight acoustic features: syllable duration, entropy variance, pitch, entropy, frequency modulation (FM), pitch variance, mean frequency and pitch goodness.

For quantitative evaluation of diurnal acoustic dynamics of juveniles (*n* = 17, 45–53 phd, mean = 49.7 phd), testosterone‐implanted juveniles (*n* = 10, 44–51 phd, mean = 46.9 phd) and adults (*n* = 12, 123–512 phd, mean = 198.3 phd), 500 syllables were sampled from the first 0–1, 3–4 and 8–10 hr after the birds started singing and were analysed. To measure shifts in syllable acoustics between each time point, probability density distributions were derived for each acoustic feature using plot (density) function with the default setting of the statistics software r program ver. 2.15.2 (Ihaka & Gentleman, 1996). For calculation of syllable acoustic shifts (%) of morning (comparison between 0–1 and 3–4 hr after singing started) and afternoon (comparison between 3–4 and 8–10 hr after singing started) periods, areas in probability density distributions that were not overlaid by two time points were measured. For syllable trajectory plots (shown in Figures 1c and 5b,d), entropy variance or pitch of each syllable was plotted against generated time (left panel) or daily generated order of syllables (right panel). Song clusters were defined as continuous singing without 15‐min silence period (right panel). The *p* values for comparison of acoustic shifts between morning and afternoon periods during juvenile singing were obtained by applying linear mixed‐effects model ANOVA (fixed effect factor = time, random effect factor = individuals) with Bonferroni correction. We used restricted maximum likelihood for likelihood estimation. To calculate how the model was improved by addition of factors to the model (the *p* value), we compared the models by ANOVA. The *p* values for comparisons of acoustic shifts (%) during morning period among normal juveniles, T‐implanted juveniles and normal adults were obtained by applying the unpaired *t* test with Bonferroni correction.

### 2.3 *In‐situ* hybridization

Male zebra finches were split into 10 experimental groups: (a) 30‐min silence after light‐on (*n* = 5, 50–55 phd, mean = 53.4 phd); (b) 30‐min singing after the initiation of singing as morning singing (*n* = 12, 45–54 phd, mean = 48.7 phd); (c) 3‐hr free singing + 1‐hr silence + 30‐min singing as afternoon singing (*n* = 8, 43–60 phd, mean = 51.8 phd); (d) 7‐hr free singing + 1‐hr silence + 30‐min singing as evening singing (*n* = 8, 45–55 phd, mean = 50.5 phd); (e) 8‐hr silence after light‐on + 30‐min singing as diurnal singing‐prevented (*n* = 9, 45–54 phd, mean = 49.7 phd); (f) 7‐hr free singing + 1‐hr silence (*n* = 4, 45–55 phd, mean = 52.0 phd); (g) testosterone‐implanted + 30‐min singing after the initiation of singing (*n* = 12, 43–53 phd, mean = 47.6 phd); (h) blank‐implanted + 30‐min singing after the initiation of singing (*n* = 9, 45–51 phd, mean = 47.4 phd); (i) adult 30‐min silent/singing (*n* = 17, 103–512 phd, mean = 153 phd); and (j) early‐deafened + 30‐min singing after the initiation of singing (*n* = 6, 102–134 phd, mean = 116.5 phd). Each bird was individually placed in a sound‐attenuating box overnight, and singing behaviour (undirected singing) was recorded after lights on. All birds which we used started singing within 30 min after lights on. For silent conditions, birds were inhibited from singing by manually tapping cages when they started singing. For brain sampling, the birds were subsequently killed by decapitation. Brains were removed and immediately embedded in OCT compound (Sakura Fine Tech, Tokyo, Japan) inside tissue block moulds, frozen on dry ice and stored at −80°C until use. Singing duration was defined as the total time of singing during the last 30 min before decapitation for brain sampling.

To clone the partial cDNA of *Arc* (1,607 bp), PCR was performed on cDNA synthesized from total RNA from adult male zebra finch brains with primers (For: 5′‐ATTCAAGGTGCTGAGAGC‐3′, Rev: 5′‐TTGCAGCAGATATTTCAAAG‐3′). PCR products were ligated into pGEM‐T Easy plasmid (Promega, Madison, WI, USA) and sequenced. *Arc* cDNA fragments with T7 and Sp6 promoter sites were PCR amplified with M13 forward and reverse primers from the inserted pGEM‐T Easy plasmid. The amplified PCR fragments were used as DNA template for in vitro transcription using T7 RNA polymerase (Roche, Basel, Switzerland) to generate the antisense ^35^S‐UTP‐labelled *Arc* riboprobes.

Frozen sections (12‐μm thick) were cut in the sagittal or coronal plane. Brain sections for a given experiment were simultaneously fixed in 3% paraformaldehyde/1× PBS (pH 7.4), washed in 1× PBS, acetylated, dehydrated in ascending ethanol series, air‐dried and processed for in situ hybridization with antisense ^35^S‐UTP labelled *Arc* riboprobes. A total of 1 × 10^6^ cpm of the ^35^S‐probe was added to a hybridization solution (50% formamide, 10% dextran, 1× Denhardt's solution, 12 mm EDTA (pH8.0), 10 mm Tris‐HCl (pH8.0), 300 mm NaCl, 0.5 mg/ml yeast tRNA and 10 mm dithiothreitol). Hybridization was performed at 67°C for 13 hr. The slides were washed in 2× SSPE and 0.1% β‐mercaptoethanol at room temperature for 1 hr, 2× SSPE, 50% formamide and 0.1% β‐mercaptoethanol at 67°C for 1 hr and 0.1× SSPE twice at 67°C for 30 min each. Slides were dehydrated in ascending ethanol series and exposed to X‐ray film (Biomax MR, Kodak, Rochester, NY, USA) for 24 hr to avoid overexposure of signal. The slides were then dipped in an autoradiographic emulsion (NTB2; Kodak), incubated for 2 weeks and processed with D‐19 developer (Kodak) and fixer (Kodak). Developed slides were Nissl‐stained with Cresyl violet acetate solution (Sigma, St Louis, MO, USA) for the capture of high‐resolution images.

For quantification of *Arc* mRNA signal, brain images on exposed X‐ray films were taken with a microscope (Z16 APO; Leica, Wetzlar, Germany) connected to a CCD camera (DFC490; Leica) with application suite V3 imaging software (Leica) (Mori & Wada, 2015). To minimize handling bias for signal detection among experimental groups, we hybridized the relevant samples at the same time for each experimental comparison and exposed them on the same sheet of X‐ray films. The same light settings were used for all images. Photoshop (Adobe Systems, San Jose, CA, USA) was used to measure the mean pixel intensities in the brain areas of interest after conversion to 256 greyscale images. For comparison of the *Arc* induction rate among experimental groups, we performed an analysis of covariance (ANCOVA) to examine the homoscedasticity from the regression line of each group using *Arc* mRNA signal intensity on X‐ray films and singing duration in the last 30 min before brain sampling.

### 2.4 Testosterone administration

Each bird was anesthetized by intraperitoneal injection of pentobarbital (6.48 mg/ml; 60 μl/10 g body weight). Birds were subcutaneously implanted with a silastic tube (inner diameter, 1.0 mm; outer diameter, 2.0 mm; and length, 10 mm) (Silascon SH 100‐0N; Kaneka, Osaka, Japan) containing either crystalline testosterone (4‐Androstan‐17β‐ol‐3‐one, 1.0–1.5 mg/animal) (Wako, Osaka, Japan) [testosterone (T)‐implanted; *n* = 14] or silicon (blank‐implanted; *n* = 9) from 30 phd. After surgery, birds were placed on a heat pad in a cage until they recovered to start eating and drinking. To measure the serum testosterone of T‐implanted juveniles and blank‐implanted juveniles, blood was sampled from the carotid artery when birds were killed by decapitation for brain sampling. Sampling of brains and blood was performed by 9 a.m. after lights were turned on at 8 a.m. at 43–53 phd. Serum testosterone was quantified using a testosterone enzyme‐linked immunosorbent assay kit (Enzo, Farmingdale, NY, USA).

### 2.5 Deafening operation

We used the same set of brain samples and song files of early‐deafened zebra finches reported in a previous study (Mori & Wada, 2015). Juvenile zebra finches (17–23 phd) were deafened by cochlear extirpation. The birds were anesthetized by intraperitoneal injection of pentobarbital (6.48 mg/ml; 60 μl/10 g of body weight). After fixing the head on a custom‐made stereotaxic apparatus with ear bars, a small window was made through the neck muscle and the skull near the end of the elastic extension of the hyoid bone. A small hole was then made in the cochlear dome. The cochlea was pulled out with a fine hooked wire. Removed cochleae were confirmed by visual inspection under a dissecting microscope. After surgery, birds were placed on a heat pad in a cage until they recovered and started producing calls. Thereafter, birds were put back in their nests and kept with their parents and siblings until 32–41 phd. After fledgling, birds were kept in a breeding cage together. They were killed after 30‐ to 60‐min singing in the morning as young adults (*n* = 6, 102–134 phd, mean = 116.5 phd). As a control group, normal zebra finches of similar age (*n* = 6, 104–147 phd, mean = 130.8 phd) were killed under the same condition.

### 2.6 Statistical analysis

**Figure 3:**
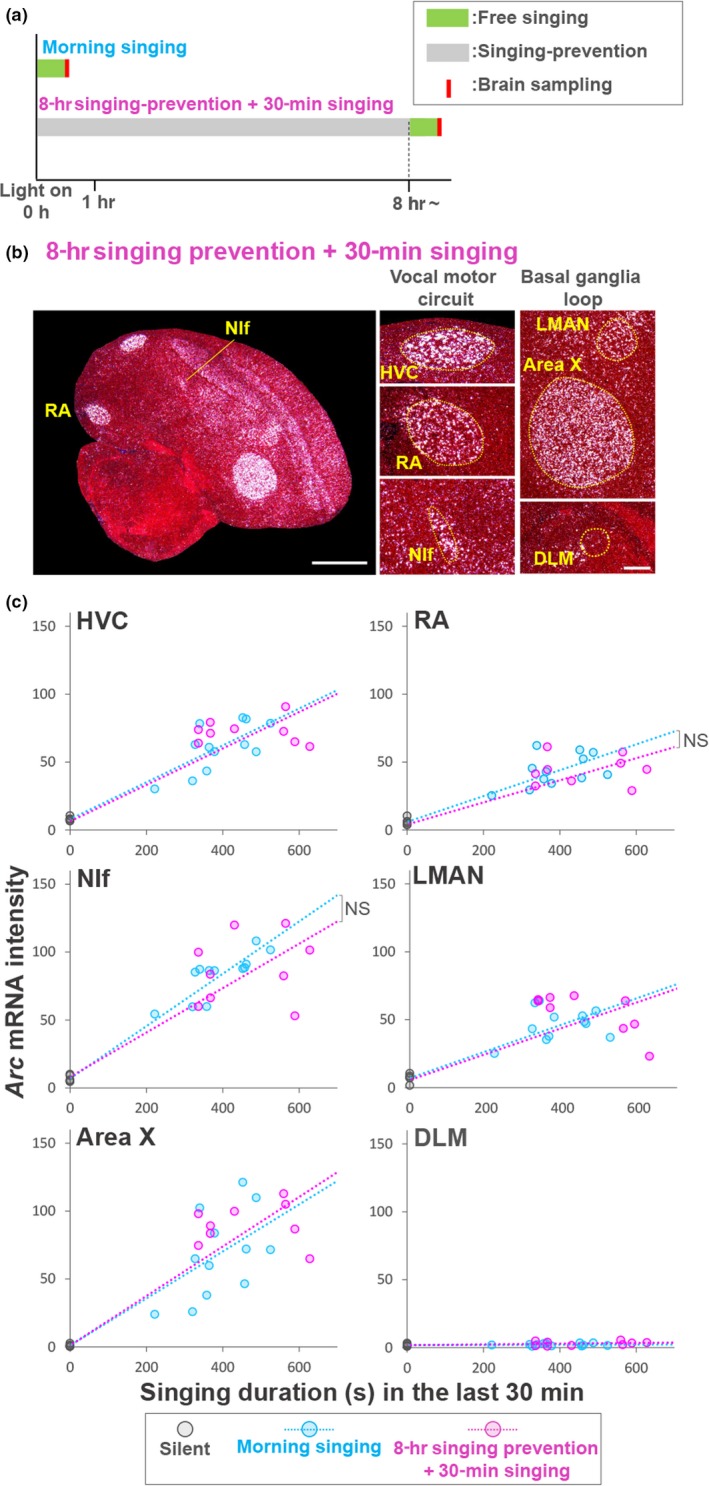
Cumulative singing practice, not circadian rhythm, associated with change in *Arc* induction rate in a day. (a) Experimental paradigm of brain sampling to test singing experience‐dependent regulation of *Arc* induction rate. (b) A typical example of induction of *Arc* mRNA after 30‐min free singing following 8‐hr singing prevention (50 phd). Singing duration (s) is shown at the bottom. Scale bar = 1.5 mm (left panel) and 200 μm (right panels). (c) Induction rate of *Arc* mRNA during morning singing (light blue; *n* = 12 birds, 45–54 phd, mean = 48.7 phd) and free singing in the afternoon after 8‐hr singing prevention (red; *n* = 9 birds, 45–54 phd, mean = 49.7 phd). Silent condition (black; *n* = 5 birds, 50–55 phd, mean = 53.4 phd). ^NS^ *p* > 0.1; ANCOVA. [Colour figure can be viewed at http://wileyonlinelibrary.com]

**Figure 4:**
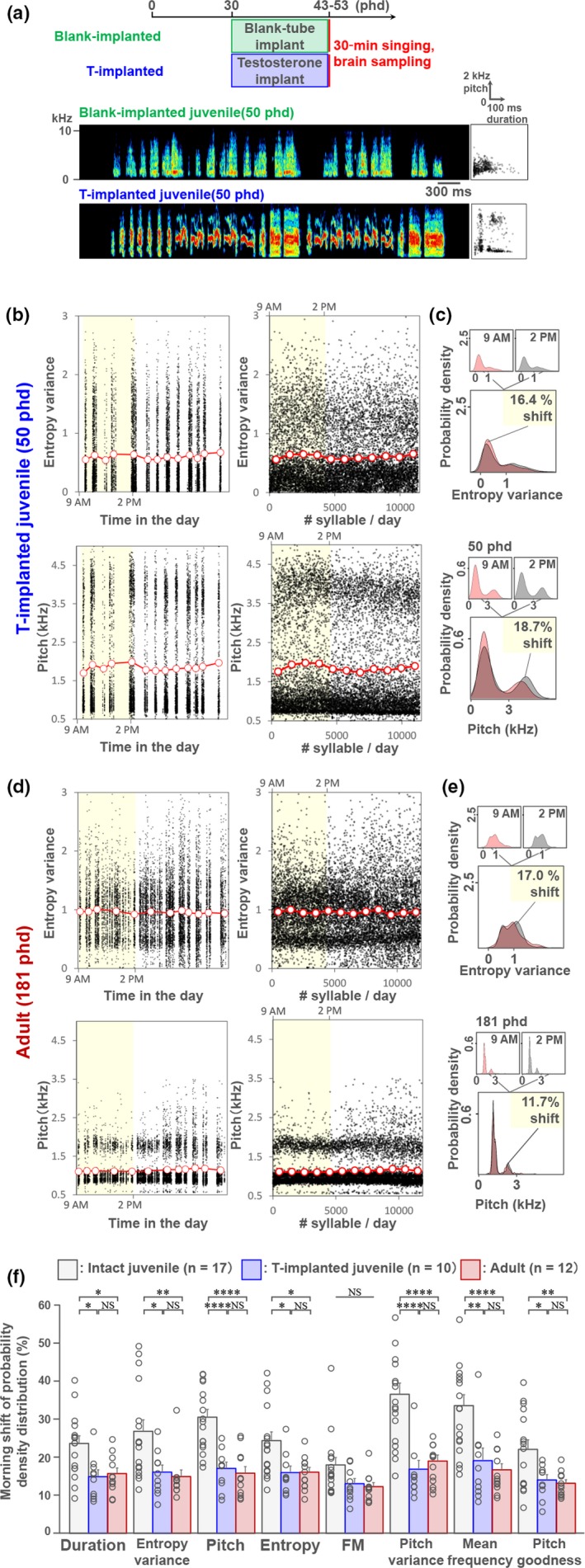
Accelerated syllable acoustic stabilization at the early plastic song stage by exogenous testosterone administration. (a) (upper panel) Experimental timeline of testosterone (T) implant and brain sampling. (lower panel) Song spectrograms of blank‐ and T‐implanted juveniles at 50 phd. (b) Diurnal trajectory plots of entropy variance and pitch of all song syllables produced by a T‐implanted juvenile (50 phd, 11,367 syllables). Acoustic features were plotted against time (left panels) or the order of syllables (right panels). Red‐lined circles indicate the average of each song cluster (left panels) or each 1,000 syllables (right panels). (c) Distribution of probability density of entropy variance and pitch in the morning (9 a.m.) and afternoon (2 p.m.) using 500 syllables at each time point (upper panels). Comparison of probability density of the two acoustic features for assessment of the acoustic shifts (%) between morning and afternoon periods (lower panels). (d) Diurnal trajectory plots of entropy variance and pitch of all song syllables produced by adult (181 phd, 11,864 syllables). (e) Distribution of probability density of entropy variance and pitch at morning (9 a.m.) and afternoon (2 p.m.) using 500 syllables at each time point (upper panels). (f) Comparison of acoustic shifts (%) during morning period between intact (*n* = 17 birds) and T‐implanted (*n* = 10 birds) juveniles, and adults (*n* = 12 birds). *p <* *0.05, **p <* *0.01, ****p <* *0.0001; unpaired *t* test with Bonferroni correction. [Colour figure can be viewed at http://wileyonlinelibrary.com]

Acoustic shifts (%) during morning and afternoon periods were compared using mixed‐model ANOVA with Bonferroni correction (Figure 1f). Acoustic shifts (%) during morning were compared among juveniles, T‐implanted birds and adults using one‐way ANOVA with Bonferroni correction followed by unpaired *t* test with Bonferroni correction as post hoc test (Figure 4f). Singing‐driven *Arc* induction was analysed using ANCOVA with Bonferroni correction (Figure 2d, 3c, 5b). *Arc* expression intensity and singing duration were compared using Mann–Whitney *U* test (Fig. 6c,d, Supporting Information Figure S1).

## 3 RESULTS

### 3.1 Diurnal variability and stabilization of syllable acoustics during the early plastic song production

To evaluate the diurnal vocal plasticity during song development, we first examined how syllable acoustics shifted in a day during the early plastic song phase (Figure 1b). We traced the trajectories of two acoustic features, entropy variance and pitch, of all syllables produced by a juvenile during two successive days (Figure 1c). The average of these two acoustic features greatly shifted during the first 3 hr after the initiation of singing and remained relatively stable dur.ing the afternoon period until sleep (Figure 1c). Although previous studies focused on a single acoustic feature, entropy variance (Deregnaucourt et al., 2005; Shank & Margoliash, 2009), it remains unclear whether other acoustic features are similarly and simultaneously regulated along with diurnal song development. We then compared the distribution shifts of eight acoustic features of syllable during morning (9 a.m. vs. 12 p.m.) and afternoon (12 p.m. vs. 7 p.m.) periods to evaluate the diurnal dynamics of syllable acoustics (Figure 1d). Although each juvenile modified individually a unique set of syllable acoustic features at different changing rates during diurnal singing, seven of examined eight acoustic features showed significantly larger shifts during the morning than during the afternoon (Figure 1e,f). This result indicates that a majority of syllable acoustics are altered mainly during the first 3 hr of singing, and the altered features are maintained until sleep during the early plastic song phase.

### 3.2 Diurnal change in singing‐driven *Arc* induction in RA and NIf

To elucidate the potential relationship between diurnal syllable acoustic plasticity and neuroplasticity‐related genes in the song circuits, we examined the induction of singing activity‐driven *Arc* at different time points of the day: morning, afternoon and evening periods during the juvenile stage (Figure 2b). Singing activity‐driven *Arc* expression is regulated in the song nuclei to peak at 30 min after the initiation of singing and then decreased during diurnal singing in adults (Wada et al., 2006). For accurate estimation of the induction response of *Arc*, we performed brain sampling at each diurnal period just after 30‐min singing before the decline in *Arc* mRNA. For brain sampling in the afternoon and evening, we allowed birds to produce spontaneous singing from morning and kept them silent for more than 1 hr to ensure the clearance of previously accumulated *Arc* mRNA (Supporting Information Figure S1) and then collected brains at 30 min after the initiation of singing at each time point (Figure 2b). We then compared the singing‐driven induction rate of *Arc* mRNA among morning, afternoon and evening periods. The induction rate was defined as induced *Arc* expression per singing amount during 30 min before brain sampling, which was calculated as *Arc* mRNA intensity divided by singing duration (seconds) in the last 30 min. First, we confirmed that the mean of singing duration in the last 30 min was not significantly different between morning, afternoon and evening singers (Supporting Information Figure S2). As a result, in the song nucleus HVC, LMAN and Area X, *Arc* mRNA was consistently induced by singing at similar induction rates at each time point of the day (Figure 2c,d). In contrast, in RA and NIf, *Arc* induction was significantly dampened in the afternoon and evening compared with its morning level (Bonferroni‐corrected ANCOVA: morning: afternoon (RA), *F*(1,24) = 26.98, *p *=* *8.7e‐5; morning: evening (RA), *F*(1,24) = 19.85, *p *=* *4.0e‐4; morning: afternoon (NIf), *F*(1,24) = 50.53, *p *=* *9.2e‐7; morning: evening (NIf), *F*(1,24) = 32.24, *p *=* *1.5e‐5) (Figure 2c,d). This result indicates that singing‐driven *Arc* induction rates in RA and NIf are different between in the first 3 hr after singing onset and other diurnal times during the early plastic song phase.

To further identify the potential regulatory mechanisms for the diurnal decrease in singing‐driven *Arc* induction during the early plastic song phase, we examined the effect of circadian rhythm on *Arc* mRNA induction in RA and NIf. For this, we prevented birds from singing for 8 hr after lights on and then allowed them to freely sing for 30 min during the evening (Figure 3a). Following free singing, the singing‐prevented juveniles intensely induced *Arc* in RA and NIf even at evening period, with similar induction rates during morning singing (Figure 3b,c). This result indicates that circadian rhythm does not causally regulate the singing‐driven *Arc* expression in RA and NIf.

### 3.3 T implant‐induced song stabilization is accompanied with a decrease in *Arc* expression in RA and NIf

**Figure 5:**
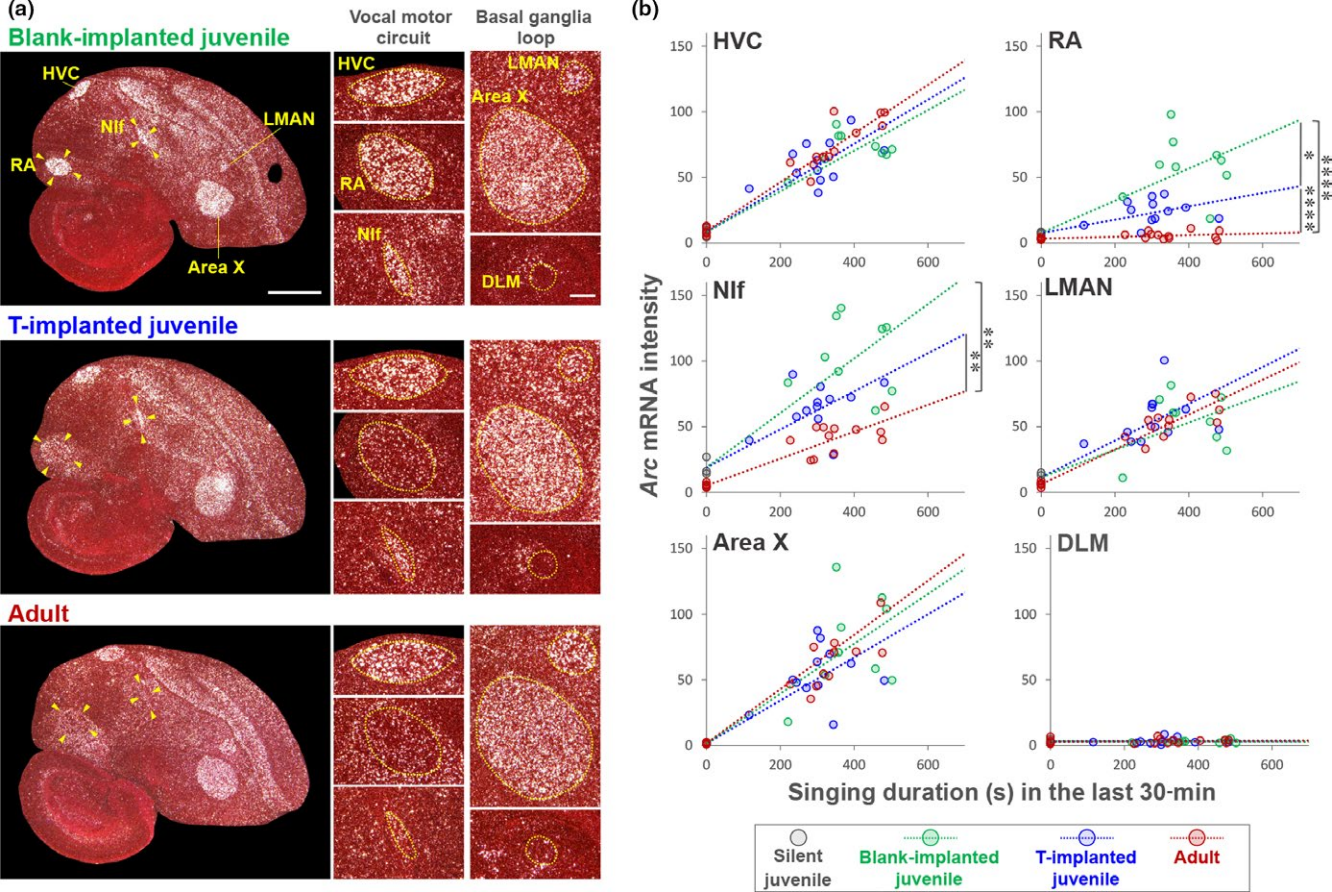
Reduced singing‐driven *Arc* induction rate in the song‐stabilized juvenile by testosterone administration. (a) *Arc* mRNA induction in blank‐ and testosterone‐implanted juveniles and adults. Singing duration (s) in 30 min after the initiation of singing is shown at the bottom. Scale bar = 1.5 mm and 200 μm. (b) Induction rate of *Arc* mRNA in song nuclei during singing at morning in blank‐implanted juveniles (green; *n* = 9 birds, 45–51 phd, mean = 47.4 phd) and T‐implanted juveniles (blue; *n* = 12 birds, 43–53 phd, mean = 47.6 phd), and adults (red; *n* = 17 birds, 103–512 phd, mean = 153 phd). Silent juveniles (*n* = 3 birds, 48–53 phd, mean = 50.3 phd) *p <* *0.01, **p <* *0.001; ANCOVA with Bonferroni correction. [Colour figure can be viewed at http://wileyonlinelibrary.com]

To further elucidate the association between syllable acoustic plasticity and *Arc* induction, we implanted T in juveniles to accelerate syllable acoustic stabilization at an earlier developmental stage than sham‐operated (blank‐implanted) juveniles (Korsia & Bottjer, 1991; Sizemore & Perkel, 2011). T implantation caused a significant increase in circulating T levels compared with blank implants (T‐implanted: 10.7 ± 1.3 ng/ml, *n* = 11 birds and blank‐implanted: 0.95 ± 0.68 ng/ml, *n* = 9 birds, respectively; unpaired *t* test, *p *=* *6.0 e‐13). As speculated, the mean shifts of syllable acoustic features in the morning were significantly reduced in T‐implanted juveniles at 43–53 phd compared with intact birds at the same age (Figure 4a–c and f). This reduced acoustic shift by T administration in juveniles resulted in a stabilized acoustic pattern that was similar to that observed in normal adult birds (Figure 4d,e,f). In the T‐implanted juveniles compared to blank‐implanted birds, singing‐driven *Arc* induction was significantly decreased in RA (Bonferroni‐corrected ANCOVA: RA, *F*(1,23) = 9.97, *p *=* *0.013) (Figure 5). The T administration‐induced decrease in the *Arc* induction rate in NIf was milder than in RA. In accordance with this association of song stabilization and attenuation of *Arc* induction rate, *Arc* induction by morning singing in normal adults was significantly decreased in RA and NIf compared with intact and T‐implanted juveniles (Bonferroni‐corrected ANCOVA: blank‐implanted juvenile: adult (RA), *F*(1,28) = 34.8, *p *=* *8.2 e‐6, blank‐implanted juvenile : adult (NIf), *F*(1,28) = 16.17, *p *=* *0.0013) (Figure 5b). These results suggest a strong correlation between vocal acoustic plasticity and *Arc* induction in RA and NIf. Furthermore, the intense induction of *Arc* after singing may be limited under low serum T level condition through development, that is from subsong to early plastic song phases.

### 3.4 Lack of relationship between *Arc* expression and song instability in early‐deafened adults

**Figure 6:**
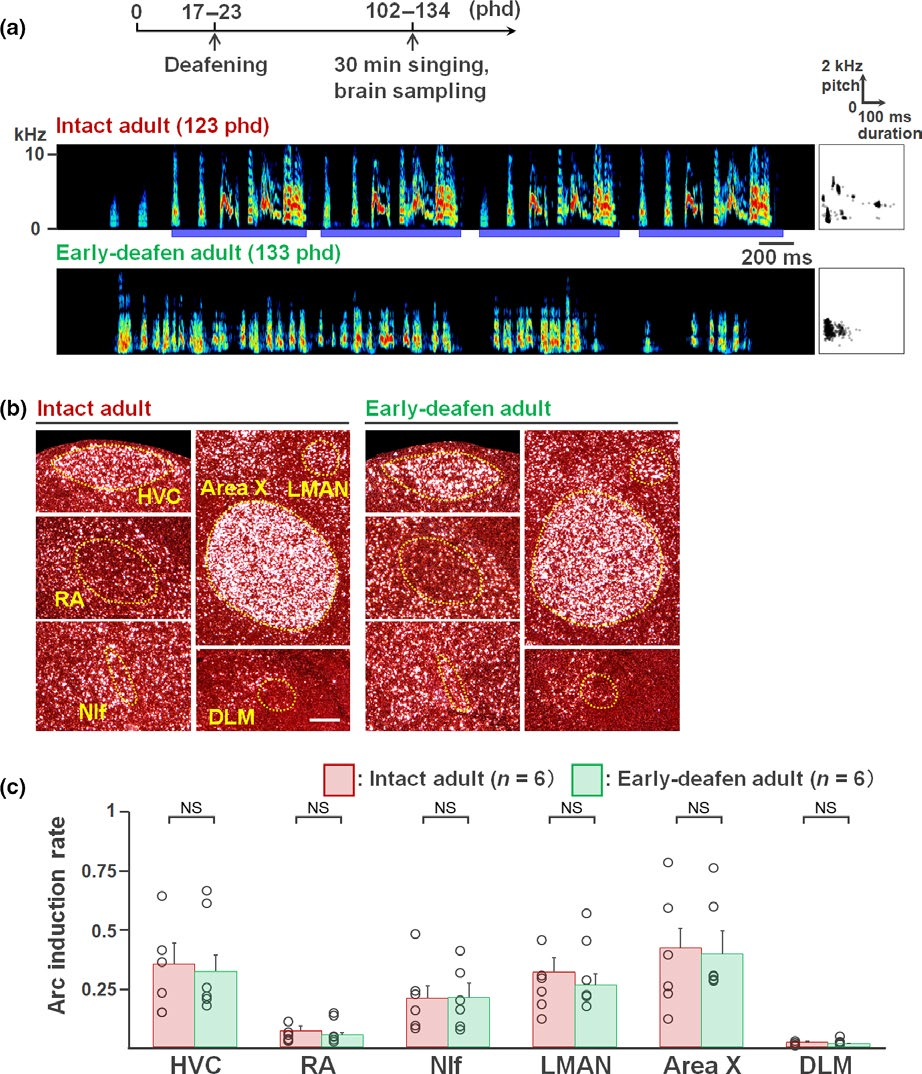
singing‐driven *Arc* expression in early‐deafened birds producing unstable songs. (a) (Upper panel) Experimental timeline for brain sampling of early‐deafened birds. (Lower panels) A typical song spectrogram of an intact adult bird (123 phd) and early‐deafened adult (133 phd). Scatter plot: distribution of 500 syllables (duration vs. pitch). (b) Singing‐driven *Arc* expression in the song nuclei in an intact adult (526 s singing) and an early‐deafened adult (645 s singing). Scale bar: 200 μm. (c) *Arc* induction rate in each song nucleus in intact (*n* = 6, 104–147 phd, mean = 130.8 phd) and early‐deafened adults (*n* = 6, 102–134 phd, mean = 116.5 phd) in the last 30‐min singing. *p *>* *0.1, Mann–Whitney *U* test. Error bars = *SEM*. [Colour figure can be viewed at http://wileyonlinelibrary.com]

To examine the potential contributions of the quality of bird's own song or auditory feedback on the modification of singing‐driven *Arc* expression in RA and NIf, we compared singing‐driven *Arc* induction between early‐deafened adults that produced unstable songs (*n* = 6, 102–134 phd, mean = 116.5 phd) and intact age‐matched adults that sing stable crystallized songs (*n* = 6, 104–147 phd, mean = 130.8 phd) (Figure 6a). However, there was no significant difference in *Arc* induction rate after 30 min of morning singing in the song nuclei including RA and NIf between early‐deafened and intact adult birds (*p *>* *0.1, Mann–Whitney *U* test). This result suggests that *Arc* expression was not modified with the quality of produced bird's own song or auditory feedback.

## 4 DISCUSSION

In this study, we aimed to elucidate the potential molecular mechanisms underlying diurnal vocal plasticity during song learning, similar to the diurnal change in motor skill improvement observed in many motor learning processes. Male zebra finches at the early plastic song phase did not continuously develop their songs during the day. Juvenile birds dramatically and simultaneously shifted multiple acoustic features of syllables during morning singing and the altered features remained relatively stable during the afternoon until the end of the day (Figure 1). In similarly aged birds, induction of the neuroplasticity‐related gene *Arc* was differently regulated in RA and NIf at different time points in a day. The expression in RA and NIf was attenuated during the first 3 hr after the initiation of singing, independent of circadian rhythm (Figure 3). The singing experience‐associated attenuation of *Arc* induction response was not observed in other song nuclei, HVC, Area X and LMAN, in which *Arc* was consistently induced at a stable rate by diurnal singing (Figure 2). The induction rates in RA and NIf were reduced when stabilized song is produced, which occurs with ageing or testosterone administration (Figures 4 and 5). However, the reduced *Arc* expression was not re‐induced by production of unstable song at adult stages (Figure 6).

### 4.1 Brain region‐specific *Arc* expression

The diurnal attenuation of *Arc* expression in RA and NIf is accompanied with cumulative singing at least for 3 hr in a day. In contrast, the reduction in *Arc* induction is not observed in HVC, LMAN and Area X in the day. Furthermore, the attenuation of *Arc* induction rates in NIf in the T‐implanted juveniles and adults was relatively mild compared with the one in RA (Figure 5). These results suggest the existence of brain region‐specific mechanisms for regulation of singing‐driven *Arc* expression even between RA and NIf. During singing at both juvenile and adult stages, robust neuronal activation is generated in the song nuclei including HVC, RA and NIf for vocal output (Goldberg & Fee, 2010; Okubo, Mackevicius, Payne, Lynch, & Fee, 2015; Ölveczky, Andalman, & Fee, 2005; Ölveczky, Otchy, Goldberg, Aronov, & Fee, 2011; Vyssotski et al., 2016). Therefore, the singing‐driven different *Arc* expression among song nuclei may not be solely controlled by neuronal activity itself.

A potential mechanism for the regulation of *Arc* expression level may involve changes in the rate of *Arc* mRNA degradation rather than changes in the transcriptional induction. However, there are several studies showing spatiotemporal dynamics of *Arc* mRNAs at one cell resolution (Guzowski, McNaughton, Barnes, & Worley, 1999; Lin et al., 2014), suggesting RNA degradation could not make a critical impact on *Arc* expression at 30‐min time point after stimuli. In those studies, after neuronal stimulation by seizure or song playback, newly transcribed *Arc* mRNAs were exported to the cytoplasm at around 15 min and still retained there at 30 min without signal attenuation. In line with these studies, in the adult zebra finch, singing activity‐driven *Arc* expression is regulated in the song nuclei to peak at 30 min after the initiation of singing and decreased during later singing in the day (Wada et al., 2006). Therefore, our brain sampling procedure at 30 min after singing initiation could avoid RNA degradation effect on *Arc* expression. However, we do not have other supporting data to explain the *Arc* expression modification specifically occurring in RA and NIf. Further studies focusing on activity‐dependent epigenetic regulation and/or developmental‐specific neurotransmitter modification will be necessary to elucidate the brain region‐specific regulation of *Arc* expression.

### 4.2 Potential causal factors for modification of singing‐driven *Arc* expression in juveniles

Is *Arc* expression modified by auditory feedback produced song quality, cumulative singing amount or other factors? First, several lines of study do not support the auditory feedback effect on *Arc* expression in the song nuclei. Auditory signals are generally attenuated or absent in the song nuclei in awake zebra finches (Cardin & Schmidt, 2004; Dave, Yu, & Margoliash, 1998; Schmidt & Konishi, 1998). In addition, neurons in the song nuclei including HVC and NIf that respond to auditory input during singing in the zebra finch there have not been not found, despite extensive investigations (Kozhevnikov & Fee, 2007; Leonardo, 2004; Vyssotski et al., 2016). In this study, we could not find significant differences of activity‐dependent *Arc* expression in the song nuclei between audition‐deprived and intact birds after singing (Figure 6). Therefore, these results suggest that *Arc* expression in the song nuclei including RA and NIf is driven by the motor action of singing, not by the repeated auditory exposure to bird's own song.

Second, a possible contribution of the quality of produced song to the modification of *Arc* expression could be considered. *Arc* expression might be strongly triggered by the production of more variable song. This possibility was partially supported by decrease in *Arc* induction in RA and NIf in T‐implanted juveniles that sang accelerated stabilized songs (Figure 5). However, the comparison between intact and early‐deafened adult birds after singing did not agree with this idea, due to no significant differences of *Arc* expression between the two groups those produced different quality of songs (stable crystallized in normal adults vs. unstable variable songs in early‐deafened adults) (Figure 6). However, both experiments cannot exclude additional effects of hormone or ageing on *Arc* induction. To segregate potential effects of the song quality and cumulative singing amount on *Arc* expression, an experiment examining *Arc* expression in deafened juveniles during afternoon singing may be crucial. Diurnal syllable acoustic shift is significantly decreased in early‐deafened compared with intact juveniles (Ohgushi et al., 2015). Therefore, when early‐deafened juveniles freely sing until afternoon period, they accumulate total diurnal singing amount but their song quality is still highly variable due to no auditory feedback. In the experiment, if *Arc* induction rate is decreased, accumulated singing amount but not reduction in song variability could be regarded as a main causal factor to modify the singing‐driven *Arc* induction rate in juvenile birds.

### 4.3 Potential contribution of *Arc* induction in RA to the development of syllable acoustics

*Arc* is an activity‐regulated neuroplasticity‐related gene, which is translocated to dendritic synaptic sites and locally translated into functional protein (Steward et al., 1998). Arc protein interacts with the endocytotic proteins endophilin and dynamin and enhances the removal of AMPARs from the postsynaptic membrane (Chowdhury et al., 2006). The removal of AMPAR from the postsynaptic sites is a crucial step for the induction of LTD (Man et al., 2000; Wang & Linden, 2000). Therefore, the molecular function of *Arc* is considered to involve the induction of protein translation‐dependent synaptic LTD (Jakkamsetti et al., 2013; Park et al., 2008; Plath et al., 2006).

RA neurons induce LTD especially at the early plastic song phase in the zebra finch (Sizemore & Perkel, 2011). RA neurons use both AMPA‐ and NMDA‐type glutamate receptors at the HVC‐RA synapse (Stark & Perkel, 1999). Therefore, singing‐driven *Arc* could regulate the removal of AMPARs from postsynaptic sites at HVC‐RA connection, which could be a critical step for the regulation of LTD in RA. In support of this idea, testosterone administration abolishes juvenile‐specific LTD (Sizemore & Perkel, 2011) and concomitantly decreases *Arc* induction rate in RA (Figure 5). Furthermore, LTD is associated with reduction in the number of dendritic spine in rat hippocampus (Bosch & Hayashi, 2012; Zhou, Homma, & Poo, 2004). In line with this, during the early‐sensorimotor learning phase in the zebra finch, HVC‐RA synapses, not LMAN‐RA synapses, are selectively refined to decrease the number of total synapses by pruning of dendritic spines (Garst‐Orozco, Babadi, & Ölveczky, 2014). Our results may suggest the possibility that the capacity of LTD and structural plasticity of dendritic spines in RA neurons are differently regulated during a day by cumulative singing experience during the early‐sensorimotor learning period. A causal study is necessary to examine the potential relationships among singing‐driven *Arc* induction in RA, synaptic plasticity and regulation of vocal acoustics.

### 4.4 Potential contribution of *Arc* induction in NIf to regulate song plasticity during the early‐sensorimotor learning phase

Although NIf is part of the auditory pathway that provides auditory input to the song nuclei HVC, auditory perturbation experiment during singing revealed that song‐related activity in NIf neurons is prevocal and does not respond to auditory error feedback for bird's own song in both juvenile and adult stages (Lewandowski & Schmidt, 2011; Vyssotski et al., 2016), indicating that neural activity in NIf under singing condition is motor but not auditory‐related. Although the developmental change in neuronal plasticity in NIf neurons has not been well elucidated, multiple lines of evidence reveal an active contribution of NIf to song learning. In zebra finch juveniles, inactivation of NIf has a drastic effect on production of plastic song causing it to return to subsong state (Naie & Hahnloser, 2011). In contrast, lesions and inactivation of NIf in adult birds lead to a transient (hours to days) disruptions in song sequence stereotypy (Cardin, Raksin, & Schmidt, 2005; Naie & Hahnloser, 2011; Otchy et al., 2015). These studies suggest that the significance of NIf contribution to song production developmentally changes during the critical period of song learning. If so, singing‐driven *Arc* induction in NIf could play a role to regulate the activity‐dependent physiological and structural changes in NIf neurons to develop song acoustics at juvenile stage. Further studies using *Arc* overexpression or downregulation techniques need to examine a causal link between *Ar*c expression in NIf and vocal plasticity during the early plastic song phase.

In conclusion, our results suggest a potential functional relationship between diurnal vocal acoustic development and changes in *Arc* induction in RA and NIf during song development. If so, this functional link may further contribute to regulate the critical period for vocal learning via *Arc*‐related synaptic plasticity. Moreover, the present results provide insight into the cumulative practice‐driven transcriptional plasticity underlying motor skill learning and development.

## DATA ACCESSIBILITY

The authors confirm that all of the data underlying the reported findings are included in the article or in supplementary data files. All raw data are available from the authors upon request.

## AUTHORS’ CONTRIBUTION

S.H. and K.W. designed the research. S.H. performed the experiments. S.H. and K.W. performed the analysis. S.H. and K.W. wrote the paper. The authors declare no competing financial interests.
