# Supplementary material for: Singing activity‐driven Arc expression associated with vocal acoustic plasticity in juvenile songbird
Source: Eur J Neurosci. 2018 Jul 6;48(2):1728–42. doi: 10.1111/ejn.14057 (PMC6099458; doi:10.1111/ejn.14057)
Supplement: Supplementary file 1 [file EJN-48-1728-s001.pdf]

Supporting information

Title

Singing activity-driven *Arc* expression associated with vocal acoustic plasticity in juvenile songbird

Shin Hayase and Kazuhiro Wada

(Supplementary Fig. 1)

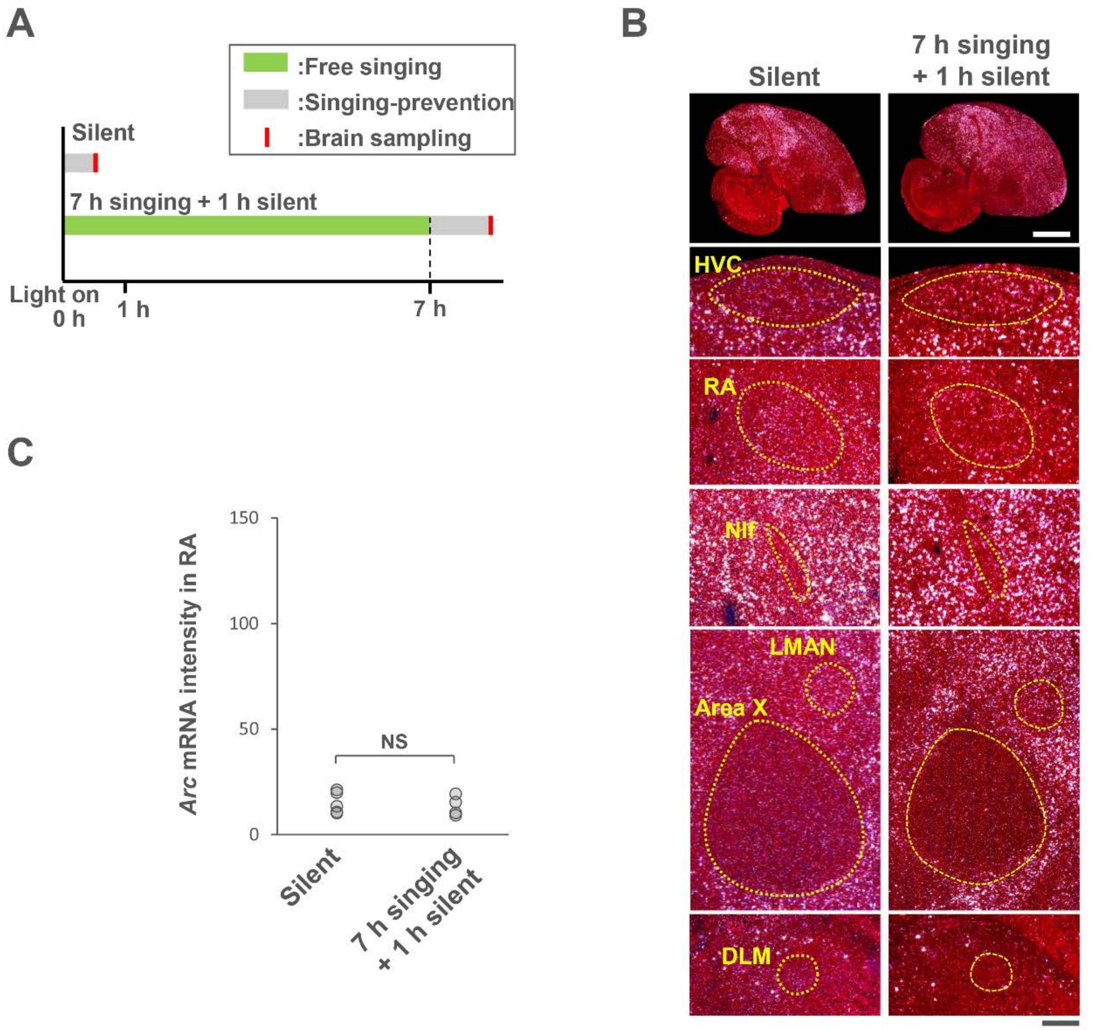

Supplementary Fig. 1.

Baseline expression of *Arc* mRNA after 1 hour silence following free singing.

(A) Diagram of experimental procedure.

(B) Baseline expression of *Arc* mRNA in song nuclei in each condition (scale bars, 1 mm and 200 μm).

(C) *Arc* mRNA expression intensity in RA in each condition (30 min silent, n = 5 birds; 1 h silent after 7 h free singing, n = 4. $p > 0.05$, Mann-Whitney U-test).

(Supplementary Fig. 2)

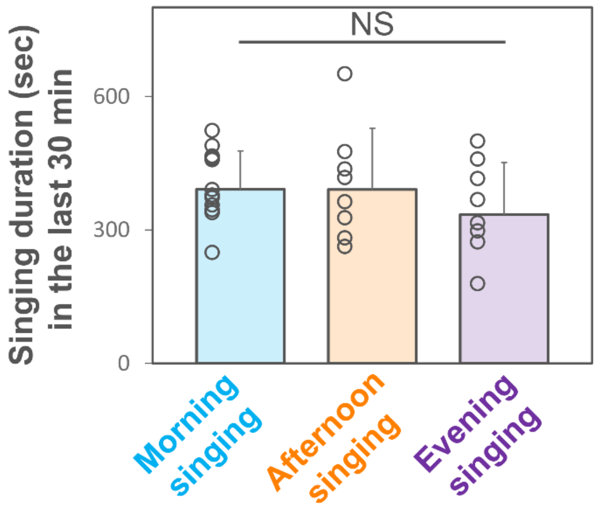

Supplementary Fig. 2.

Singing duration in the last 30 min before brain sampling for morning, afternoon, and evening singing in juvenile zebra finches

Singing duration (sec) in the last 30 min in morning (light blue, n = 12), afternoon (orange, n = 8), and evening (purple, n = 8) singing conditions. $p > 0.1$, One-way ANOVA.
